# Need for continual education about disaster medicine for health professionals in China-a pilot study

**DOI:** 10.1186/1471-2458-11-89

**Published:** 2011-02-09

**Authors:** Bo Huang, Jing Li, Yunkai Li, Weidong Zhang, Futang Pan, Shujun Miao

**Affiliations:** 1 Unit of Orthopedics, Jinan Fourth People's Hospital, Jinan, 250033, China; 2 Unit of Anesthesiology, Jinan Fourth People's Hospital, Jinan, 250033, China; 3 Office for Disease Control and Emergency Response, Chinese Center for Disease Control and Prevention, Beijing, 102206, China; 4 Institute of Dermatology, Shandong Medical Science Academy, Jinan, 250022, China; 5 Ninghe Chinese Medicine Hospital, Ninghe County, Tianjin, 301500, China

## Abstract

**Background:**

Disaster Medicine training is not included in medical education curriculum in China, even though the country has suffered various disasters annually. We intended to assess the need for continual education regarding disaster management for health professionals in China.

**Methods:**

A survey was conducted among 324 health professionals who participated in the response to the Wenchuan earthquake medical relief and public health assessment in October, 2008.

**Results:**

The most of participants (67.3%) received informal disaster medicine training, and only a few (12.7%) participated in disaster drills. Most of the participants wanted to get continual education about disaster medicine training (89.8%), but prefer on-line training course for the flexibility of time scheduling and travel through China.

**Conclusion:**

The need for continual disaster medicine training is high; health professionals should be equipped with knowledge and skills for disaster management.

## Background

China is one of the countries most affected by natural disasters; it is an important restricting factor for economic and social development [1-3]. The deadliest natural disaster in two recent decades was the Sichuan Wenchuan earthquake on 12 May 2008 - the death toll was 88,928. Additionally, floods continue to be the major mode of natural disasters in China [1,4,5].

Disaster Medicine (DM) is not included in medical education curriculum in China, even though the country suffers various disasters every year [6,7]. The Chinese Medical Association (CMA) is the largest medical organization in China, and it plays a leading and active role in the nation's medical education and professional training [8,9]. It organizes continual medical education for licensed clinical and public health doctors, and participants receive credit which is a part of physicians' annual assessment. Every doctor needs to obtain certain credits for annual qualification, however, most training is focused on clinical medicine, while public health training and disaster medicine training are overlooked. After the SARS epidemic in 2003 and Wenchuan Earthquake in 2008, public health emergency and disaster medicine training have received increasing attention [10-12].

In this study we intended to assess the need for continual education regarding disaster management for health professionals in China.

## Methods

### Study sample

This cross-sectional study was conducted in December, 2008 in Beijing, China after Wenchuan earthquake. A simple sampling method was used to recruit the samples. A list of 400 health professionals who participated in the Wenchuan earthquake medical mission was obtained from the Wenchuan earthquake logistics office with names, telephone numbers and e-mail addresses. The ethical committee of China CDC (Institutional Review Board) approved the study protocol. Anonymity and confidentiality were guaranteed to the participants, and informed consent from participants was required during the survey. The data was collected by self-administered anonymous questionnaires through paper survey by mail. Experts from the research fields of public health and disaster epidemiology reviewed the questionnaire. The questions covered the following categories: demographic information, experience with disasters, attitudes towards disaster medicine training, and source of knowledge and willingness to participate the training. Variables were dichotomized: the answers yes versus no. We collected information of attitude about the favorite forms of disaster training, including intensive formal training courses, on-line training courses, provision of training documents, and self learning.

### Data analysis

Epi Data 3.1(Epi Data for Windows; Epi Data Association, Odense, Denmark) was used to establish a database by double entry. First, data cleaning was conducted, and logic errors were not considered into the data analysis. SPSS software (version 14.0 for Windows; SPSS Inc., Chicago, IL) was used to analyze the dataset. For the analysis reported here, we categorized the variable of favourite forms of disaster training into two categories: (1) formal training for intensive course; and (2) informal training for on-line training course, only providing training documents, and self learning. Logistic regression was used to analyze independent association between need of continual DM training and sample characteristics.

## Results

A total of 400 questionnaires were sent. There were 76 questionnaires that were not returned, so the final sample size was 324 included in the analysis (a response rate of 81%). The sample was predominantly male with 13:1 male-to-female ratio. Age range was between 24 and 57 years, with a median of 35 years. There were 59.6% have received a Master's or Doctor Degree (of which 62 participants possessed a PhD degree), and Bachelor's Degree or less accounted for 40.4%. High education system is different in China, for the medical students they got MD after 5 years high education, then for a PhD diploma normally needs 5-6 years institutional research [9]. There were 16.4% (53/324) who has previously participated in disaster medical relief missions (domestic or in other countries) prior to the Wenchuan earthquake. Positive attitudes toward DM training were common, as most of the participants wanted to receive continual education for disaster medicine training (89.8%). In response to a question about the favorite training style, more participants (51.9%) prefer informal training such as on-line training courses or providing training materials for the flexibility of time scheduling and travel through China. (See Table 1).

**Table 1:** Characteristics of samples

Variables		N = 324	percent %
Gender	Male	301	92.9
	Female	23	7.1
Age	≤35	162	50.0
	>35	162	50.0
Education	Bachelor or below	131	40.4
	Master or Doctor	193	59.6
Working years	≤10 years	153	47.2
	>10 years	171	52.8
Trained for DM	Yes	218	67.3
	No	106	32.7
Drill for DM	Yes	41	12.7
	No	283	87.3
Once participated DM relief	Yes	53	16.4
	No	271	83.6
Need of continual DM training	Yes	291	89.8
	No	33	10.2
Training style priority	formal	156	48.1
	informal	168	51.9

Based on logistic regression analysis, factor significantly associated with the need of continual DM training and sample characteristics was training style priority: formal training versus informal (OR 2.35.10; 95% CI 1.03-5.35; p < 0.05) (See Table 2).

**Table 2:** Logistic regression analysis of sample characteristics associated with the need of continual DM training

Variables		OR	**95% C.I**.		*P*
				
			Lower	Upper	
Gender	female vs. male	0.44	0.14	1.36	0.153
Education background	Master or Doctor vs. Bachelor or below	1.15	0.53	2.50	0.727
Age	>35 vs. ≤35	1.03	0.27	3.92	0.964
Working years	>10 vs. ≤10	0.50	0.13	1.91	0.306
Once participated DM relief	yes vs. no	1.10	0.36	3.38	0.862
Once trained	yes vs. no	1.92	0.88	4.16	0.100
Once participated drill	yes vs. no	5.01	0.64	39.41	0.126
Favourite training style	formal vs. informal	2.35	1.03	5.35	<0.05

## Discussion

This study gives us an overview about the need for continual disaster medicine training among health professionals in China. Our finding demonstrates that the need of continual disaster medicine training is high; CMA and other related institutions should organize continual medical education on public health training and disaster medicine training to improve the research on disaster medicine and public health emergency preparedness. Providing knowledge and training of disaster epidemiology and disaster management could increase the awareness of disaster response among health professionals [13-15]. In this study, information about the knowledge on disaster medicine epidemiology and management was limited. We formulated questions about "myth and reality in disaster situations" based on the WHO's Technical Guidelines [16] - Emergency Management Essentials to assess the awareness and knowledge of disaster situations of health professional in China; however, it was not included in the analysis for the large number of incomplete and missing data.

During the survey, China had recently experienced the Wenchuan earthquake, which was the deadliest natural disaster in two recent decades. It caused energetic debates about including Disaster Medicine in the medical education curriculum, because continual training for disaster medicine is separated among public health professionals and clinical physicians in China. All participants participated the Wenchuan medical relief mission and did receive short intensive training for disaster management and risk assessment before they were sent to the after-earthquake zone. It focused on infectious diseases control, environment hygiene, risk assessment, security, and other topics. Little knowledge about disaster epidemiology and management was distributed. In fact, disaster management training is a continuous process by which all individuals, groups, and communities who are involved in learning the knowledge and tactical skills to manage hazards or ameliorate the impact of disasters resulting from the hazards [15]. Disaster medicine training covers a wide variety of disciplines; training should focus on how to take preventive and preparatory measures for future disasters, and how to use creative and innovative approaches in solving disaster challenges [13,15,17]. Training style could be flexible, as our study demonstrates most of participants prefer informal training such as on-line course for the flexibility of time scheduling. In order to enhance training effectiveness, targeted training programs in feasible forms should consider the disaster characteristics and potential hazards in China by discovering which approach could work best.

Comprehensive educational needs are increasing for those seeking DM training in this field. Medical leaders should realize the academic need for continual disaster medicine training based on the country's disaster profile with a science and knowledge-based approach [13,18].

## Conclusion

Healthcare providers in China participating in domestic disaster relief indicate the need for DM is high. This study supports the need for DM training of healthcare providers, and provides direction for further research in DM training in China.

## Competing interests

The authors declare that they have no competing interests.

## Authors' contributions

BH, JL, YL, FP, WZ and SM participated in the design of study. BH, JL acquisition of data, analysis and interpretation of data. FP, SM helped for the data analysis. WZ wrote the final version of the text. All authors read and approved the final manuscript.

## Pre-publication history

The pre-publication history for this paper can be accessed here:

http://www.biomedcentral.com/1471-2458/11/89/prepub
